# 3,5,7-Tripropyl-1-aza­adamantane-4,6,10-triol

**DOI:** 10.1107/S1600536808006284

**Published:** 2008-04-04

**Authors:** Pierre-Loïc Saaidi, Pierre-Etienne Chazal, Philippe Maurin, Erwann Jeanneau, Jens Hasserodt

**Affiliations:** a Laboratoire de Chimie, UMR CNRS 5182, Ecole Normale Supérieure de Lyon, 46 Allée d’Italie, 69364 Lyon, France; b Centre de Diffractométrie Henri Longchambon, Université Claude Bernard Lyon1, 43 boulevard du 11 novembre 1918, 69622 Villeurbanne Cedex, France

## Abstract

The title compound, C_18_H_33_NO_3_, was prepared according to a highly diastereoselective hydrogenation procedure from 3,5,7-triallyl-1-aza­adamantane-4,6,10-trione. The crystal structure of the title compound contains two crystallographically independent mol­ecules (*Z*′ = 2), which are linked by inter­molecular hydrogen bonding into chains. In contrast to the aza­adamantanones, the aza­adamantanetriol core of the title compound does not show any particular C—C bond elongation.

## Related literature

For related literature on the consequences of through-bond donor–acceptor inter­actions in β-amino­ketones azaadaman­tones, see: Lampkins *et al.* (2008 ▶). For details on mol­ecular receptors based on a polyfunctionalized rigid platform, see: Guarise *et al.* (2006 ▶); Haberhauer *et al.* (2005 ▶); Li *et al.* (2005 ▶). For information about mol­ecules displaying multiple formula units per crystallographic asymmetric unit, see: Steiner (2000 ▶). For bond lengths in similar compounds, see: Lampkins *et al.* (2008 ▶); Allen *et al.* (1987 ▶). Details on the synthesis can be found in: Risch (1985 ▶); Li *et al.* (2005 ▶). For details of data collection and refinement procedures, see: Görbitz (1999 ▶); Guarise *et al.* (2006 ▶); Prince (1982 ▶); Watkin (1994 ▶).
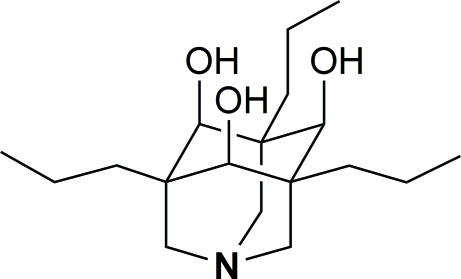


## Experimental

### 

#### Crystal data

C_18_H_33_NO_3_
*M* *_r_* = 311.45
Monoclinic, 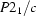

*a* = 13.1922 (2) Å
*b* = 22.6174 (5) Å
*c* = 13.1144 (3) Å
β = 114.4470 (10)°
*V* = 3562.17 (13) Å^3^
*Z* = 8
Mo *K*α radiation
μ = 0.08 mm^−1^
*T* = 150 K
0.43 × 0.35 × 0.14 mm

#### Data collection

Nonius KappaCCD diffractometer
Absorption correction: none
16642 measured reflections
8498 independent reflections
5272 reflections with *I* > 2σ(*I*)
*R*_int_ = 0.027

#### Refinement

*R*[*F* ^2^ > 2σ(*F* ^2^)] = 0.045
*wR*(*F* ^2^) = 0.054
*S* = 1.06
5272 reflections
397 parameters
H-atom parameters constrained
Δρ_max_ = 0.29 e Å^−3^
Δρ_min_ = −0.18 e Å^−3^

### 

Data collection: *COLLECT* (Nonius, 2001 ▶).; cell refinement: *DENZO*/*SCALEPACK* (Otwinowski & Minor, 1997 ▶); data reduction: *DENZO*/*SCALEPACK*; program(s) used to solve structure: *SIR97* (Altomare *et al.*, 1999 ▶); program(s) used to refine structure: *CRYSTALS* (Betteridge *et al.*, 2003 ▶); molecular graphics: *DIAMOND* (Brandenburg & Putz, 1996 ▶); software used to prepare material for publication: *CRYSTALS*.

## Supplementary Material

Crystal structure: contains datablocks global, I. DOI: 10.1107/S1600536808006284/nc2092sup1.cif

Structure factors: contains datablocks I. DOI: 10.1107/S1600536808006284/nc2092Isup2.hkl

Additional supplementary materials:  crystallographic information; 3D view; checkCIF report

## Figures and Tables

**Table 1:** Hydrogen-bond geometry (Å, °)

*D*—H⋯*A*	*D*—H	H⋯*A*	*D*⋯*A*	*D*—H⋯*A*
C26—H261⋯O4^i^	0.97	2.38	3.261 (3)	151
C35—H351⋯O4^i^	0.97	2.54	3.344 (3)	140
C31—H312⋯O1^ii^	0.97	2.54	3.355 (3)	142
O3—H3⋯N2^iii^	0.84	1.87	2.696 (3)	168
O2—H2⋯O3	0.82	2.08	2.788 (3)	143
O4—H4⋯O5	0.84	1.96	2.696 (3)	147
O5—H5⋯N1^iv^	0.83	1.90	2.713 (3)	167
O1—H1⋯O3	0.79	2.07	2.777 (3)	149
O6—H6⋯O5	0.83	2.07	2.810 (3)	148

Symmetry codes: (i) 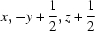; (ii) 
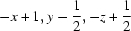
; (iii) 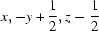; (iv) 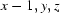.

**Fig. 1.**
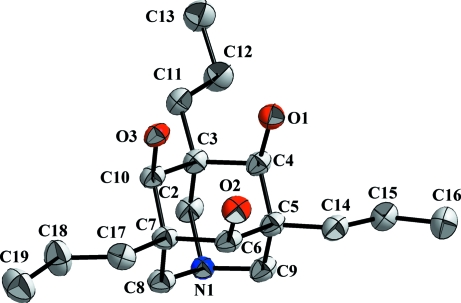
Molecule A of the title compound with displacement ellipsoids drawn at the 50% probability level. Hydrogen atoms have been omitted fo clarity.

**Fig. 2.**
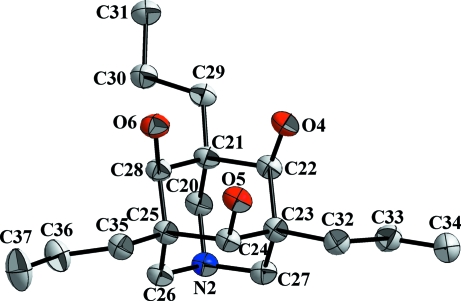
Molecule B of the title compound with displacement ellipsoids drawn at the 50% probability level. Hydrogen atoms have been omitted fo clarity.

## supplementary crystallographic information

### Comment

Recently, an increasing number of molecular receptors based on a polyfunctionalized rigid platform have been reported (Guarise  et al., 2006; Haberhauer et al., 2005 ; Li et al., 2005). Among them, adamantane and aza-adamantane scaffolds offer the guarantee of a well-defined environment as a result of axial or equatorial substituent orientation. The title compound shows a perfectly constraint face with three hydroxyl groups in axial positions that could potentially be used, after introduction of suitable ligands, as oriented binding sites for a wide range of applications.

The asymmetric unit of the title compound consists of two independent molecules(A and B) per unit-cell (Z'=2) (Steiner, 2000). This feature arises from the different orientation of one of the propyl group (C11-C12-C13 and C29-C30-C31). Contrary to the case of aza-adamantanone systems which show several C-C bond elongations (Lampkins et al., 2008), all angles and distances within the two molecules agree well with the expected values from the literature (Allen et al., 2006). Each independant molecule of the title compound displays two intra-molecular O-H···O hydrogen bonds and one O-H···N inter-molecular. The later leads to the formation of infinite one-dimensional chains along [2 0 1]. The chains are composed of alternated molecules A and B.

### Experimental

The title compound was prepared according to a hydrogenation procedure adapted from Risch (1985). A 100-ml reactor vessel was charged with 80 mg of 3,5,7-triallyl-1-aza-adamantane-4,6,10-trione (0.267 mmol) (Li *et al.*, 2005), 88 mg of platinum oxide (0.388 mmol) and 20 ml of a 1:1 THF/0.1 N HCl mixture. The reaction mixture was hydrogenated (10 bar H_2_) for 50 h at room temperature. After filtration over Celite, the aqueous solution was concentrated under reduced pressure (200 mbar) basified with potassium carbonate and extracted with four 80-ml portions of ethyl acetate. The combined organic phases were then dried over Na_2_SO_4_ and concentrated *in vacuo*. Flash chromatography purification (petroleum ether/ethyl acetate 1:1 then neat ethyl acetate) afforded the title compound as a cristalline white powder (54 mg, 65%). X-ray quality single crystals were obtained by slow evaporation from a cyclohexane/ethyl acetate solution.

### Refinement

Changes in illuminated volume were kept to a minimum, and were taken into account (Görbitz, 1999) by the multi-scan inter-frame scaling (*DENZO*/*SCALEPACK*, Otwinowski & Minor, 1997). All non hydrogen atoms were refined with anisotropic displacement parameters. The H atoms were all located in a difference map and initially refined with soft restraints on the bond lengths and angles to regularize their geometry (C—H in the range 0.954–1.014 and O—H = 0.794–0.836 Å) and *U*_iso_(H) (in the range 1.2–1.5 times *U*_eq_ of the parent atom). In the final refinement the H-atoms were refined using a riding model.

### Crystal data

**Table:**

C_18_H_33_NO_3_	*F*_000_ = 1376
*M**_r_* = 311.45	*D*_x_ = 1.162 Mg m^−^^3^
Monoclinic, *P*2_1_/*c*	Mo *K*α radiation λ = 0.71069 Å
Hall symbol: -P 2ybc	Cell parameters from 8626 reflections
*a* = 13.1922 (2) Å	θ = 0.7–27.9º
*b* = 22.6174 (5) Å	µ = 0.08 mm^−^^1^
*c* = 13.1144 (3) Å	*T* = 150 K
β = 114.4470 (10)º	Block, colorless
*V* = 3562.17 (13) Å^3^	0.43 × 0.35 × 0.14 mm
*Z* = 8	

### Data collection

**Table:**

Nonius KappaCCD diffractometer	5272 reflections with *I* > 2σ(*I*)
Monochromator: graphite	*R*_int_ = 0.027
*T* = 150 K	θ_max_ = 27.9º
φ & ω scans	θ_min_ = 1.8º
Absorption correction: none	*h* = −16→17
16642 measured reflections	*k* = −29→29
8498 independent reflections	*l* = −17→17

### Refinement

**Table:**

Refinement on *F*	Hydrogen site location: inferred from neighbouring sites
Least-squares matrix: full	H-atom parameters constrained
*R*[*F*^2^ > 2σ(*F*^2^)] = 0.045	Method, part 1, Chebychev polynomial, (Watkin, 1994, *P*rince, 1982) [*w*eight] = 1.0/[A_0_*T_0_(x) + A_1_*T_1_(x) ··· + A_n-1_]*T_n-1_(x)] where A_i_ are the Chebychev coefficients listed belo*w* and x = *F* /*F*max Method = Robust Weighting (*P*rince, 1982) W = [*w*eight] * [1-(delta*F*/6*sigma*F*)^2^]^2^ A_i_ are: 0.361 0.287 0.915E-01
*wR*(*F*^2^) = 0.054	(Δ/σ)_max_ = 0.0003
*S* = 1.06	Δρ_max_ = 0.29 e Å^−^^3^
5272 reflections	Δρ_min_ = −0.18 e Å^−^^3^
397 parameters	Extinction correction: None
Primary atom site location: structure-invariant direct methods	

### Fractional atomic coordinates and isotropic or equivalent isotropic displacement parameters (Å^2^)

**Table:**

	*x*	*y*	*z*	*U*_iso_*/*U*_eq_	
O4	0.22669 (10)	0.25151 (5)	0.14425 (9)	0.0370	
C22	0.31510 (13)	0.23591 (7)	0.24925 (13)	0.0319	
C21	0.32122 (13)	0.16842 (7)	0.27185 (13)	0.0317	
C28	0.22217 (13)	0.14597 (8)	0.29517 (13)	0.0321	
O6	0.11955 (9)	0.14471 (6)	0.19701 (9)	0.0394	
C25	0.21442 (12)	0.18139 (7)	0.39228 (13)	0.0307	
C24	0.20674 (12)	0.24822 (7)	0.36626 (13)	0.0307	
C23	0.30828 (13)	0.27030 (7)	0.34760 (13)	0.0318	
C32	0.30115 (14)	0.33789 (8)	0.32981 (15)	0.0363	
C33	0.40054 (15)	0.36720 (8)	0.31923 (16)	0.0427	
C34	0.38186 (18)	0.43335 (9)	0.29532 (17)	0.0490	
C27	0.41327 (13)	0.25457 (7)	0.45347 (14)	0.0335	
N2	0.42274 (11)	0.19088 (6)	0.47731 (11)	0.0325	
C26	0.32503 (13)	0.17096 (8)	0.49585 (13)	0.0323	
C20	0.42662 (13)	0.15834 (8)	0.38098 (13)	0.0332	
O5	0.10637 (9)	0.26078 (5)	0.26700 (9)	0.0358	
C35	0.11432 (13)	0.16295 (8)	0.41669 (14)	0.0348	
C36	0.11823 (16)	0.10271 (9)	0.47005 (18)	0.0468	
C37	0.01314 (19)	0.09196 (11)	0.4888 (2)	0.0652	
C29	0.32990 (14)	0.13606 (8)	0.17273 (14)	0.0360	
C30	0.33494 (17)	0.06849 (8)	0.17741 (15)	0.0426	
C31	0.33958 (17)	0.04267 (9)	0.07231 (16)	0.0464	
O2	0.67551 (10)	0.29792 (6)	0.42136 (11)	0.0413	
C6	0.77461 (13)	0.29360 (8)	0.40309 (14)	0.0353	
C7	0.75659 (13)	0.26000 (8)	0.29401 (14)	0.0338	
C8	0.87254 (14)	0.25041 (8)	0.29625 (15)	0.0380	
N1	0.93330 (11)	0.30585 (7)	0.30284 (12)	0.0385	
C2	0.86849 (14)	0.34194 (9)	0.20364 (14)	0.0380	
C3	0.75199 (13)	0.35754 (8)	0.19632 (13)	0.0333	
C10	0.69017 (12)	0.29876 (8)	0.19190 (13)	0.0320	
O3	0.58136 (9)	0.30951 (5)	0.18883 (9)	0.0341	
C4	0.76841 (13)	0.39174 (8)	0.30365 (13)	0.0350	
C5	0.83343 (13)	0.35359 (8)	0.40852 (14)	0.0351	
C9	0.94652 (14)	0.33854 (9)	0.40532 (14)	0.0392	
C14	0.85052 (15)	0.38593 (8)	0.51758 (14)	0.0402	
C15	0.91418 (17)	0.44422 (9)	0.54001 (16)	0.0468	
C16	0.9286 (2)	0.47099 (10)	0.65167 (17)	0.0577	
O1	0.66640 (10)	0.41465 (6)	0.30104 (10)	0.0421	
C11	0.68602 (14)	0.39337 (8)	0.08912 (14)	0.0377	
C12	0.72891 (18)	0.45457 (9)	0.08051 (17)	0.0520	
C13	0.6514 (2)	0.48575 (10)	−0.02511 (18)	0.0578	
C17	0.70163 (15)	0.19948 (8)	0.29070 (15)	0.0399	
C18	0.6838 (2)	0.16093 (9)	0.19042 (19)	0.0578	
C19	0.6477 (2)	0.09893 (10)	0.1998 (2)	0.0639	
H221	0.3844	0.2468	0.2435	0.0364*	
H281	0.2387	0.1039	0.3201	0.0372*	
H241	0.2047	0.2695	0.4312	0.0362*	
H321	0.2918	0.3557	0.3926	0.0433*	
H322	0.2344	0.3461	0.2617	0.0433*	
H331	0.4665	0.3621	0.3906	0.0517*	
H332	0.4140	0.3482	0.2579	0.0522*	
H341	0.4453	0.4515	0.2884	0.0734*	
H342	0.3710	0.4531	0.3553	0.0734*	
H343	0.3152	0.4400	0.2268	0.0738*	
H271	0.4115	0.2754	0.5181	0.0400*	
H272	0.4803	0.2669	0.4441	0.0397*	
H261	0.3231	0.1927	0.5587	0.0376*	
H262	0.3335	0.1285	0.5132	0.0369*	
H201	0.4923	0.1716	0.3704	0.0379*	
H202	0.4354	0.1162	0.4003	0.0387*	
H351	0.1073	0.1926	0.4673	0.0420*	
H352	0.0468	0.1652	0.3451	0.0413*	
H361	0.1826	0.1005	0.5424	0.0572*	
H362	0.1255	0.0714	0.4211	0.0579*	
H372	0.0181	0.0545	0.5240	0.1066*	
H373	0.0058	0.1234	0.5365	0.1065*	
H371	−0.0529	0.0913	0.4176	0.1066*	
H292	0.3977	0.1499	0.1666	0.0441*	
H291	0.2660	0.1473	0.1059	0.0432*	
H302	0.3993	0.0553	0.2435	0.0525*	
H301	0.2675	0.0528	0.1838	0.0534*	
H312	0.3437	−0.0001	0.0775	0.0710*	
H311	0.4058	0.0579	0.0642	0.0701*	
H313	0.2727	0.0538	0.0072	0.0718*	
H81	0.9151	0.2263	0.3611	0.0457*	
H82	0.8659	0.2291	0.2287	0.0451*	
H101	0.6800	0.2775	0.1226	0.0385*	
H41	0.8178	0.4270	0.3091	0.0411*	
H91	0.9852	0.3762	0.4079	0.0453*	
H92	0.9911	0.3150	0.4707	0.0451*	
H141	0.8903	0.3587	0.5797	0.0480*	
H142	0.7783	0.3931	0.5183	0.0461*	
H151	0.9877	0.4377	0.5396	0.0542*	
H152	0.8730	0.4720	0.4804	0.0538*	
H161	0.9692	0.5090	0.6648	0.0816*	
H163	0.9716	0.4448	0.7131	0.0814*	
H162	0.8570	0.4791	0.6549	0.0813*	
H112	0.6828	0.3697	0.0256	0.0445*	
H111	0.6100	0.3982	0.0830	0.0418*	
H122	0.8028	0.4512	0.0804	0.0611*	
H121	0.7358	0.4786	0.1446	0.0606*	
H132	0.6794	0.5245	−0.0329	0.0821*	
H131	0.6403	0.4627	−0.0907	0.0830*	
H133	0.5790	0.4914	−0.0244	0.0836*	
H171	0.7490	0.1778	0.3584	0.0495*	
H172	0.6303	0.2058	0.2953	0.0493*	
H181	0.7507	0.1601	0.1784	0.0724*	
H182	0.6265	0.1791	0.1236	0.0733*	
H192	0.6309	0.0768	0.1305	0.0976*	
H193	0.7068	0.0790	0.2604	0.0985*	
H191	0.5823	0.0993	0.2172	0.0980*	
H61	0.8258	0.2699	0.4657	0.0408*	
H3	0.5379	0.3125	0.1209	0.0493*	
H2	0.6248	0.3068	0.3608	0.0653*	
H4	0.1729	0.2612	0.1586	0.0565*	
H5	0.0605	0.2783	0.2841	0.0541*	
H1	0.6209	0.3901	0.2705	0.0617*	
H6	0.0936	0.1786	0.1939	0.0573*	
H21	0.9078	0.3782	0.2069	0.0497*	
H22	0.8620	0.3215	0.1358	0.0487*	

### Atomic displacement parameters (Å^2^)

**Table:**

	*U*^11^	*U*^22^	*U*^33^	*U*^12^	*U*^13^	*U*^23^
O4	0.0343 (6)	0.0458 (7)	0.0311 (6)	0.0050 (5)	0.0137 (5)	0.0050 (5)
C22	0.0273 (7)	0.0388 (9)	0.0293 (8)	0.0020 (6)	0.0116 (6)	0.0026 (6)
C21	0.0291 (8)	0.0384 (8)	0.0284 (7)	0.0031 (6)	0.0126 (6)	0.0005 (6)
C28	0.0279 (7)	0.0374 (8)	0.0287 (7)	−0.0001 (6)	0.0093 (6)	0.0001 (6)
O6	0.0324 (6)	0.0493 (7)	0.0323 (6)	−0.0034 (5)	0.0091 (5)	−0.0039 (5)
C25	0.0259 (7)	0.0370 (8)	0.0297 (8)	0.0015 (6)	0.0119 (6)	0.0009 (6)
C24	0.0263 (7)	0.0367 (8)	0.0288 (7)	0.0024 (6)	0.0113 (6)	0.0015 (6)
C23	0.0285 (7)	0.0350 (8)	0.0323 (8)	0.0013 (6)	0.0128 (6)	0.0010 (6)
C32	0.0349 (8)	0.0369 (9)	0.0381 (9)	0.0024 (7)	0.0163 (7)	0.0005 (7)
C33	0.0433 (10)	0.0418 (10)	0.0456 (10)	−0.0037 (8)	0.0210 (8)	−0.0041 (8)
C34	0.0589 (12)	0.0426 (10)	0.0478 (10)	−0.0065 (9)	0.0245 (9)	0.0020 (8)
C27	0.0267 (7)	0.0394 (9)	0.0326 (8)	0.0008 (6)	0.0105 (6)	−0.0015 (7)
N2	0.0265 (6)	0.0394 (7)	0.0308 (7)	0.0020 (5)	0.0111 (5)	0.0001 (6)
C26	0.0282 (7)	0.0392 (9)	0.0288 (8)	0.0032 (6)	0.0110 (6)	0.0016 (6)
C20	0.0278 (8)	0.0410 (9)	0.0314 (8)	0.0048 (6)	0.0130 (6)	0.0011 (6)
O5	0.0264 (5)	0.0481 (7)	0.0328 (6)	0.0075 (5)	0.0121 (5)	0.0040 (5)
C35	0.0289 (8)	0.0404 (9)	0.0352 (8)	0.0007 (7)	0.0135 (7)	0.0011 (7)
C36	0.0419 (10)	0.0494 (11)	0.0528 (11)	0.0029 (8)	0.0233 (9)	0.0110 (9)
C37	0.0587 (13)	0.0608 (14)	0.0930 (18)	−0.0020 (11)	0.0483 (13)	0.0190 (13)
C29	0.0375 (9)	0.0402 (9)	0.0331 (8)	0.0017 (7)	0.0175 (7)	−0.0010 (7)
C30	0.0536 (11)	0.0412 (10)	0.0369 (9)	0.0001 (8)	0.0228 (8)	−0.0020 (7)
C31	0.0568 (11)	0.0452 (10)	0.0413 (10)	−0.0021 (9)	0.0245 (9)	−0.0061 (8)
O2	0.0349 (6)	0.0527 (8)	0.0416 (6)	0.0001 (5)	0.0211 (5)	0.0007 (6)
C6	0.0289 (8)	0.0454 (9)	0.0318 (8)	0.0045 (7)	0.0127 (6)	0.0035 (7)
C7	0.0295 (8)	0.0390 (9)	0.0341 (8)	0.0035 (7)	0.0145 (6)	0.0015 (7)
C8	0.0308 (8)	0.0448 (10)	0.0388 (9)	0.0054 (7)	0.0148 (7)	0.0018 (7)
N1	0.0295 (7)	0.0484 (9)	0.0376 (7)	0.0019 (6)	0.0139 (6)	−0.0005 (6)
C2	0.0308 (8)	0.0489 (10)	0.0359 (9)	−0.0027 (7)	0.0156 (7)	0.0004 (7)
C3	0.0295 (8)	0.0387 (8)	0.0323 (8)	−0.0026 (7)	0.0133 (6)	0.0001 (7)
C10	0.0252 (7)	0.0399 (9)	0.0320 (8)	0.0005 (6)	0.0129 (6)	−0.0005 (6)
O3	0.0246 (5)	0.0460 (7)	0.0304 (5)	0.0011 (5)	0.0103 (4)	0.0000 (5)
C4	0.0285 (8)	0.0433 (9)	0.0323 (8)	−0.0009 (7)	0.0118 (6)	−0.0004 (7)
C5	0.0282 (8)	0.0445 (9)	0.0314 (8)	0.0007 (7)	0.0110 (6)	0.0005 (7)
C9	0.0291 (8)	0.0518 (11)	0.0344 (8)	0.0001 (7)	0.0108 (7)	0.0000 (7)
C14	0.0353 (9)	0.0511 (11)	0.0324 (8)	0.0014 (8)	0.0121 (7)	−0.0009 (7)
C15	0.0441 (10)	0.0503 (11)	0.0395 (9)	−0.0010 (8)	0.0108 (8)	−0.0031 (8)
C16	0.0637 (13)	0.0567 (13)	0.0444 (11)	−0.0011 (10)	0.0141 (10)	−0.0097 (9)
O1	0.0347 (6)	0.0481 (7)	0.0407 (6)	0.0053 (5)	0.0127 (5)	−0.0058 (6)
C11	0.0360 (9)	0.0427 (9)	0.0318 (8)	−0.0043 (7)	0.0115 (7)	0.0005 (7)
C12	0.0532 (11)	0.0482 (11)	0.0472 (11)	−0.0104 (9)	0.0133 (9)	0.0067 (9)
C13	0.0705 (14)	0.0475 (12)	0.0478 (11)	−0.0071 (10)	0.0170 (10)	0.0088 (9)
C17	0.0400 (9)	0.0391 (9)	0.0432 (9)	0.0016 (7)	0.0200 (8)	0.0034 (7)
C18	0.0787 (15)	0.0433 (11)	0.0572 (13)	−0.0092 (10)	0.0340 (12)	−0.0053 (9)
C19	0.0616 (14)	0.0473 (12)	0.0867 (17)	−0.0052 (10)	0.0344 (13)	−0.0071 (12)

### Geometric parameters (Å, °)

**Table:**

O4—C22	1.4323 (19)	O2—C6	1.427 (2)
O4—H4	0.836	O2—H2	0.824
C22—C21	1.551 (2)	C6—C7	1.549 (2)
C22—C23	1.540 (2)	C6—C5	1.550 (2)
C22—H221	0.979	C6—H61	0.979
C21—C28	1.546 (2)	C7—C8	1.533 (2)
C21—C20	1.545 (2)	C7—C10	1.536 (2)
C21—C29	1.537 (2)	C7—C17	1.541 (2)
C28—O6	1.4311 (19)	C8—N1	1.471 (2)
C28—C25	1.543 (2)	C8—H81	0.970
C28—H281	1.000	C8—H82	0.980
O6—H6	0.833	N1—C2	1.473 (2)
C25—C24	1.544 (2)	N1—C9	1.480 (2)
C25—C26	1.546 (2)	C2—C3	1.541 (2)
C25—C35	1.540 (2)	C2—H21	0.962
C24—C23	1.541 (2)	C2—H22	0.974
C24—O5	1.4498 (18)	C3—C10	1.548 (2)
C24—H241	0.988	C3—C4	1.541 (2)
C23—C32	1.543 (2)	C3—C11	1.542 (2)
C23—C27	1.543 (2)	C10—O3	1.4400 (19)
C32—C33	1.526 (2)	C10—H101	0.987
C32—H321	0.969	O3—H3	0.839
C32—H322	0.978	C4—C5	1.548 (2)
C33—C34	1.528 (3)	C4—O1	1.429 (2)
C33—H331	0.986	C4—H41	1.014
C33—H332	0.990	C5—C9	1.548 (2)
C34—H341	0.970	C5—C14	1.538 (2)
C34—H342	0.965	C9—H91	0.986
C34—H343	0.974	C9—H92	0.973
C27—N2	1.468 (2)	C14—C15	1.525 (3)
C27—H271	0.978	C14—H141	0.982
C27—H272	0.982	C14—H142	0.970
N2—C26	1.477 (2)	C15—C16	1.522 (3)
N2—C20	1.481 (2)	C15—H151	0.983
C26—H261	0.969	C15—H152	0.975
C26—H262	0.982	C16—H161	0.990
C20—H201	0.979	C16—H163	0.972
C20—H202	0.981	C16—H162	0.981
O5—H5	0.829	O1—H1	0.794
C35—C36	1.523 (3)	C11—C12	1.517 (3)
C35—H351	0.974	C11—H112	0.976
C35—H352	0.992	C11—H111	0.979
C36—C37	1.525 (3)	C12—C13	1.513 (3)
C36—H361	0.978	C12—H122	0.978
C36—H362	0.987	C12—H121	0.973
C37—H372	0.954	C13—H132	0.974
C37—H373	0.978	C13—H131	0.964
C37—H371	0.979	C13—H133	0.967
C29—C30	1.530 (3)	C17—C18	1.513 (3)
C29—H292	0.981	C17—H171	0.980
C29—H291	0.965	C17—H172	0.977
C30—C31	1.521 (3)	C18—C19	1.502 (3)
C30—H302	0.976	C18—H181	0.960
C30—H301	0.992	C18—H182	0.980
C31—H312	0.969	C19—H192	0.980
C31—H311	0.986	C19—H193	0.964
C31—H313	0.973	C19—H191	0.979
			
C22—O4—H4	106.1	C6—O2—H2	106.8
O4—C22—C21	112.55 (13)	O2—C6—C7	112.81 (14)
O4—C22—C23	111.96 (13)	O2—C6—C5	114.11 (14)
C21—C22—C23	110.71 (13)	C7—C6—C5	110.76 (13)
O4—C22—H221	106.2	O2—C6—H61	105.0
C21—C22—H221	106.7	C7—C6—H61	107.1
C23—C22—H221	108.5	C5—C6—H61	106.4
C22—C21—C28	112.32 (13)	C6—C7—C8	106.33 (14)
C22—C21—C20	106.14 (13)	C6—C7—C10	109.84 (14)
C28—C21—C20	106.18 (13)	C8—C7—C10	107.59 (13)
C22—C21—C29	108.86 (13)	C6—C7—C17	110.63 (14)
C28—C21—C29	112.08 (14)	C8—C7—C17	109.20 (14)
C20—C21—C29	111.09 (13)	C10—C7—C17	112.98 (14)
C21—C28—O6	113.01 (13)	C7—C8—N1	113.30 (14)
C21—C28—C25	110.05 (13)	C7—C8—H81	107.9
O6—C28—C25	113.04 (13)	N1—C8—H81	108.9
C21—C28—H281	106.8	C7—C8—H82	109.7
O6—C28—H281	105.8	N1—C8—H82	108.4
C25—C28—H281	107.7	H81—C8—H82	108.6
C28—O6—H6	104.0	C8—N1—C2	108.51 (13)
C28—C25—C24	110.28 (13)	C8—N1—C9	109.61 (14)
C28—C25—C26	106.56 (12)	C2—N1—C9	109.42 (14)
C24—C25—C26	107.18 (13)	N1—C2—C3	112.43 (14)
C28—C25—C35	112.72 (13)	N1—C2—H21	109.2
C24—C25—C35	109.14 (13)	C3—C2—H21	108.2
C26—C25—C35	110.79 (13)	N1—C2—H22	110.1
C25—C24—C23	111.76 (13)	C3—C2—H22	110.1
C25—C24—O5	110.02 (13)	H21—C2—H22	106.6
C23—C24—O5	108.96 (12)	C2—C3—C10	107.58 (14)
C25—C24—H241	108.0	C2—C3—C4	107.37 (13)
C23—C24—H241	108.3	C10—C3—C4	109.76 (13)
O5—C24—H241	109.7	C2—C3—C11	110.44 (13)
C22—C23—C24	108.97 (13)	C10—C3—C11	109.11 (13)
C22—C23—C32	113.24 (14)	C4—C3—C11	112.46 (14)
C24—C23—C32	110.03 (13)	C7—C10—C3	110.85 (13)
C22—C23—C27	107.17 (13)	C7—C10—O3	108.86 (13)
C24—C23—C27	107.33 (13)	C3—C10—O3	111.07 (13)
C32—C23—C27	109.90 (13)	C7—C10—H101	109.6
C23—C32—C33	116.31 (14)	C3—C10—H101	108.8
C23—C32—H321	107.7	O3—C10—H101	107.6
C33—C32—H321	108.2	C10—O3—H3	106.2
C23—C32—H322	107.5	C3—C4—C5	110.40 (14)
C33—C32—H322	108.5	C3—C4—O1	112.65 (13)
H321—C32—H322	108.3	C5—C4—O1	113.17 (14)
C32—C33—C34	111.86 (16)	C3—C4—H41	107.6
C32—C33—H331	108.5	C5—C4—H41	105.8
C34—C33—H331	108.3	O1—C4—H41	106.8
C32—C33—H332	109.5	C4—C5—C6	111.37 (13)
C34—C33—H332	109.1	C4—C5—C9	106.53 (14)
H331—C33—H332	109.6	C6—C5—C9	106.02 (14)
C33—C34—H341	111.2	C4—C5—C14	112.05 (15)
C33—C34—H342	110.6	C6—C5—C14	109.69 (14)
H341—C34—H342	107.8	C9—C5—C14	110.97 (14)
C33—C34—H343	110.3	C5—C9—N1	112.46 (14)
H341—C34—H343	109.5	C5—C9—H91	107.4
H342—C34—H343	107.3	N1—C9—H91	109.1
C23—C27—N2	112.55 (13)	C5—C9—H92	109.6
C23—C27—H271	109.6	N1—C9—H92	109.1
N2—C27—H271	108.5	H91—C9—H92	109.1
C23—C27—H272	109.9	C5—C14—C15	116.59 (15)
N2—C27—H272	107.7	C5—C14—H141	107.2
H271—C27—H272	108.5	C15—C14—H141	108.6
C27—N2—C26	109.33 (12)	C5—C14—H142	108.8
C27—N2—C20	109.62 (13)	C15—C14—H142	108.6
C26—N2—C20	109.22 (13)	H141—C14—H142	106.6
C25—C26—N2	112.30 (13)	C14—C15—C16	111.66 (17)
C25—C26—H261	108.8	C14—C15—H151	109.6
N2—C26—H261	108.5	C16—C15—H151	109.5
C25—C26—H262	109.2	C14—C15—H152	108.9
N2—C26—H262	108.3	C16—C15—H152	108.7
H261—C26—H262	109.8	H151—C15—H152	108.4
C21—C20—N2	112.41 (13)	C15—C16—H161	110.9
C21—C20—H201	109.5	C15—C16—H163	110.6
N2—C20—H201	108.2	H161—C16—H163	107.3
C21—C20—H202	110.3	C15—C16—H162	112.2
N2—C20—H202	107.7	H161—C16—H162	106.9
H201—C20—H202	108.5	H163—C16—H162	108.7
C24—O5—H5	110.4	C4—O1—H1	105.5
C25—C35—C36	117.98 (14)	C3—C11—C12	117.40 (15)
C25—C35—H351	106.7	C3—C11—H112	107.4
C36—C35—H351	107.3	C12—C11—H112	108.8
C25—C35—H352	107.5	C3—C11—H111	107.8
C36—C35—H352	109.1	C12—C11—H111	107.0
H351—C35—H352	108.0	H112—C11—H111	108.1
C35—C36—C37	110.91 (17)	C11—C12—C13	111.20 (16)
C35—C36—H361	109.9	C11—C12—H122	109.4
C37—C36—H361	108.6	C13—C12—H122	109.3
C35—C36—H362	109.7	C11—C12—H121	110.5
C37—C36—H362	109.3	C13—C12—H121	108.5
H361—C36—H362	108.4	H122—C12—H121	107.9
C36—C37—H372	109.6	C12—C13—H132	111.9
C36—C37—H373	109.0	C12—C13—H131	111.1
H372—C37—H373	110.0	H132—C13—H131	108.5
C36—C37—H371	111.0	C12—C13—H133	110.4
H372—C37—H371	107.7	H132—C13—H133	107.5
H373—C37—H371	109.6	H131—C13—H133	107.2
C21—C29—C30	117.32 (14)	C7—C17—C18	116.01 (15)
C21—C29—H292	107.9	C7—C17—H171	107.6
C30—C29—H292	107.2	C18—C17—H171	108.2
C21—C29—H291	107.4	C7—C17—H172	108.8
C30—C29—H291	107.9	C18—C17—H172	109.3
H292—C29—H291	108.9	H171—C17—H172	106.7
C29—C30—C31	111.38 (15)	C17—C18—C19	114.05 (19)
C29—C30—H302	110.4	C17—C18—H181	109.4
C31—C30—H302	109.9	C19—C18—H181	109.8
C29—C30—H301	109.5	C17—C18—H182	108.6
C31—C30—H301	108.3	C19—C18—H182	108.2
H302—C30—H301	107.3	H181—C18—H182	106.5
C30—C31—H312	110.0	C18—C19—H192	111.1
C30—C31—H311	109.5	C18—C19—H193	109.1
H312—C31—H311	109.2	H192—C19—H193	108.8
C30—C31—H313	109.5	C18—C19—H191	110.5
H312—C31—H313	108.9	H192—C19—H191	109.8
H311—C31—H313	109.7	H193—C19—H191	107.6

### Hydrogen-bond geometry (Å, °)

**Table:**

*D*—H···*A*	*D*—H	H···*A*	*D*···*A*	*D*—H···*A*
C26—H261···O4^i^	0.97	2.38	3.261 (3)	151
C35—H351···O4^i^	0.97	2.54	3.344 (3)	140
C31—H312···O1^ii^	0.97	2.54	3.355 (3)	142
O3—H3···N2^iii^	0.84	1.87	2.696 (3)	168
O2—H2···O3	0.82	2.08	2.788 (3)	143
O4—H4···O5	0.84	1.96	2.696 (3)	147
O5—H5···N1^iv^	0.83	1.90	2.713 (3)	167
O1—H1···O3	0.79	2.07	2.777 (3)	149
O6—H6···O5	0.83	2.07	2.810 (3)	148

Symmetry codes: (i) *x*, −*y*+1/2, *z*+1/2; (ii) −*x*+1, *y*−1/2, −*z*+1/2; (iii) *x*, −*y*+1/2, *z*−1/2; (iv) *x*−1, *y*, *z*.
